# Coordinated response of milk bacterial and metabolic profiles to subacute ruminal acidosis in lactating dairy cows

**DOI:** 10.1186/s40104-023-00859-8

**Published:** 2023-05-04

**Authors:** Yingyu Mu, Wangpan Qi, Tao Zhang, Jiyou Zhang, Shengyong Mao

**Affiliations:** 1 Ruminant Nutrition and Feed Engineering Technology Research Center, College of Animal Science and Technology, Nanjing Agricultural University, Nanjing, 210095 China; 2 Laboratory of Gastrointestinal Microbiology, Jiangsu Key Laboratory of Gastrointestinal Nutrition and Animal Health, National Center for International Research on Animal Gut Nutrition, College of Animal Science and Technology, Nanjing Agricultural University, Nanjing, 210095 China

**Keywords:** High-concentrate diet, Milk bacteria, Milk metabolome, Milk quality, Subacute ruminal acidosis

## Abstract

**Background:**

Bovine milk is an important source of nutrition for human consumption, and its quality is closely associated with the microbiota and metabolites in it. But there is limited knowledge about the milk microbiome and metabolome in cows with subacute ruminal acidosis.

**Methods:**

Eight ruminally cannulated Holstein cows in mid lactation were selected for a 3-week experiment. The cows were randomly allocated into 2 groups, fed either a conventional diet (CON; 40% concentrate; dry matter basis) or a high-concentrate diet (HC; 60% concentrate; dry matter basis).

**Results:**

The results showed that there was a decreased milk fat percentage in the HC group compared to the CON group. The amplicon sequencing results indicated that the alpha diversity indices were not affected by the HC feeding. At the phylum level, the milk bacteria were dominated by Proteobacteria, Actinobacteria, Bacteroidetes, and Firmicutes both in the CON and HC groups. At the genus level, the HC cows displayed an improved proportion of *Labrys* (*P* = 0.015) compared with the CON cows. Results of both the principal components analysis and partial least squares of discriminant analysis of milk metabolome revealed that samples of the CON and HC groups clustered separately. A total of 31 differential metabolites were identified between the two groups. Of these, the levels of 11 metabolites decreased (α-linolenic acid, prostaglandin E2, *L*-lactic acid, *L*-malic acid, 3-hydroxysebacic acid, succinyladenosine, guanosine, pyridoxal, *L*-glutamic acid, hippuric acid, and trigonelline), whereas the levels of the other 20 metabolites increased in the HC group with respect to the CON group (*P* < 0.05).

**Conclusion:**

These results suggested that subacute ruminal acidosis less impacted the diversity and composition of milk microbiota, but altered the milk metabolic profiles, which led to the decline of the milk quality.

**Supplementary Information:**

The online version contains supplementary material available at 10.1186/s40104-023-00859-8.

## Background

As an important food source for human consumption, bovine milk contains high levels of nutrients such as proteins, fatty acids, phospholipids, vitamins, and minerals [1], and can be further processed into dairy products such as cream, butter, yogurt, ice cream, and cheese [2]. With the improvement of people’s living standards, the demand for milk and dairy products is gradually increasing. However, forage often does not contain enough energy to support the high milk production, and therefore high-concentrate (HC) diets are widely used in modern dairy production, which conversely leads to a high incidence of subacute ruminal acidosis (SARA) [3–5].

Previous studies revealed that cows might develop mastitis during a grain-based SARA challenge, accompanied by the alteration of microbial composition in the milk [6, 7]. The microbiota composition of raw milk is an important consideration for mammary gland health [8]. Moreover, the microbiota count is one of the most important quality indicators related to the milk price. In Germany and other European countries, the microbial load of class 1 raw milk should be not more than 100,000 CFU/mL according to the European Regulation No 853/2004 [9]. In the United States, the microbial load of grade ‘A’ milk should be less than 100,000 CFU/mL according to the Pasteurized Milk Ordinance [10].

Besides milk fat, milk protein, and milk lactose, the milk also contains a variety of different small-molecule metabolites and micronutrients [11]. These metabolites are primarily derived from the metabolism of the mammary epithelial cells [12], and can be used as the mirror of the mammary gland function and milk quality [13]. As mentioned above, SARA could impact the health of mammary gland, which might further affect its metabolism. However, limited knowledge is available on the changes in the milk metabolites during SARA.

Therefore, the objectives of our study were to explore the bacterial changes and metabolic profiles in the milk of cows with SARA by using the 16S rRNA gene sequencing and high-performance liquid chromatography-mass spectrometry (HPLC-MS)-based metabolomics analyses, and finally, to clarify the effects of SARA on mammary gland health and milk quality in lactating dairy cows.

## Methods

### Animals, diets, and experimental design

Eight healthy multiparous lactating Holstein cows (2 to 3 parity) with an initial body weight of 582 ± 50 kg were chosen for the experiment, and were housed in individually tethered stalls with good ventilation. On average, the cows were in 120 ± 6 d in milk, with a mean milk yield of 18.2 ± 2.66 kg/d at the beginning of the experiment. All the cows were healthy and had free access to clean water throughout the 3-week experiment period. The cows were randomly allocated into two groups and fed either a conventional (CON; 40% concentrate; DM basis; *n* = 4) diet or a HC (60% concentrate; DM basis; *n* = 4) diet (Table 1). The diets had the same CP content and were formulated to meet or exceed the energy and milk production requirements of the cows according to NRC (2001) [14]. The diet was supplied twice daily at 08:00 and 19:00, with approximately 10% feed refusal. The cows were milked twice daily before feeding using a pipeline milking system.

**Table 1:** Ingredients and nutrients composition of the conventional diet (CON) and the high-concentrate diet (HC)

Item	CON	HC
Ingredients, % of DM		
Corn grain	19.40	24.92
Soybean	13.50	13.48
Barley	–	12.00
DDGS^a^	3.80	5.91
CaCO_3_	0.80	1.48
Ca(HCO_3_)_2_	1.10	0.92
NaCl	0.40	0.37
Premix^b^	1.00	0.92
Corn silage	12.00	6.00
American alfalfa hay	24.00	17.00
Australian oaten hay	24.00	17.00
Nutrients composition		
DM, %	46.77	48.03
CP, % of DM	16.16	16.12
Crude fat, % of DM	3.05	3.05
NDF, % of DM	36.14	29.92
NFC^c^, % of DM	38.68	46.04
Starch, % of DM	17.96	27.82
Ash, % of DM	5.97	4.87
Ca, % of DM	1.14	1.18
P, % of DM	0.52	0.51
NE_L_^d^, Mcal/kg of DM	1.57	1.64
NFC/NDF	0.93	1.54

^a^Dried distillers grains with solubles

^b^Premix contained the following ingredients per kilogram of diet: vitamin A, 45,000 KIU/kg; vitamin D_3_, 10,000 KIU/kg; vitamin E, 75 KIU/kg; vitamin K_3_, 10,000 mg/kg; Mn, 25,385 mg/kg; Zn, 44,769 mg/kg; Cu, 10,240 mg/kg; and Fe, 36,325 mg/kg

^c^NFC = 100 – (%NDF + %CP + %ether extract + %ash)

^d^Calculated based on Ministry of P. R. China recommendations [15]

### Sample collection

The measurement of ruminal pH and dry matter intake (DMI) were described previously [16].

Milk production was recorded on the last 2 d of each week and milk samples were collected on the last day of each week for each cow. Before sample collection, teats were first dipped in iodine and followed by physical scrubbing with alcohol. Besides, the first 2 streams of milk were discarded to minimize possible contamination. Samples were immediately divided into two portions after collection. The first portion was added with potassium dichromate tablets (milk preservative) and mixed proportionally according to the milk yield of the morning and evening; then, the composite samples were stored at 4 °C for subsequent milk composition (milk fat, milk protein, and lactose) measurements using infrared analysis with a spectrophotometer (Foss-4000, Foss Electric A/S, Hillerød, Denmark). The second portion was immediately put into liquid nitrogen until the subsequent 16S rRNA sequencing and metabolomics analysis.

### Microbial DNA isolation, 16S rRNA sequencing, and analyses

For each cow, 1–2 mL composite milk samples of morning and evening on the last day of each week were used for DNA isolation. After being fully vortexed, the milk samples were centrifuged at 13,000 × *g* for 10 min at 4 °C to remove the fat layer firstly [17]. Genomic DNA of the milk samples was extracted using a Water DNA Kit (Omega Bio-Tek, Norcross, GA, USA) according to the manufacturer’s protocols. The concentration and quality of the extracted DNA was assessed by a NanoDrop 1000 spectrophotometer (Thermo Fisher Scientific, Madison, USA) and the 1.0% agarose gel electrophoresis, respectively. All the extracted DNA samples were stored at −80 °C until subsequent processing.

The 16S rRNA genes were amplified using the 341F-806R primers (341F: 5′- CCTAYGGGRBGCASCAG-3′, 806R: 5′- GGACTACNNGGGTATCTAAT-3′) targeted the V3–V4 hypervariable region. The PCR mixtures consisted of 0.5 U of Taq polymerase (TransGen Biotech, Beijing, China) in 25 μL of 10 × PCR buffer, 200 μL each dNTP, 0.2 μmol/L each primer, and 2 μL of DNA (50 ng/μL). The amplification program was executed as follows: 95 °C for 2 min, 25 cycles at 95 °C for 30 s, 55 °C for 30 s, and 72 °C for 30 s, and a final extension at 72 °C for 5 min. We then visualized the PCR products using a 2% agarose gel, and bands between 400 and 450 bp were excised and next purified using the GeneJET Gel Extraction Kit (Thermo Scientific, Waltham, MA, USA). The paired-end sequencing (2 × 250 bp) was accomplished using an Illumina MiSeq PE 250 platform (Illumina Inc., San Diego, CA, USA) following standard Illumina sequencing protocols.

**Fig. 1:**
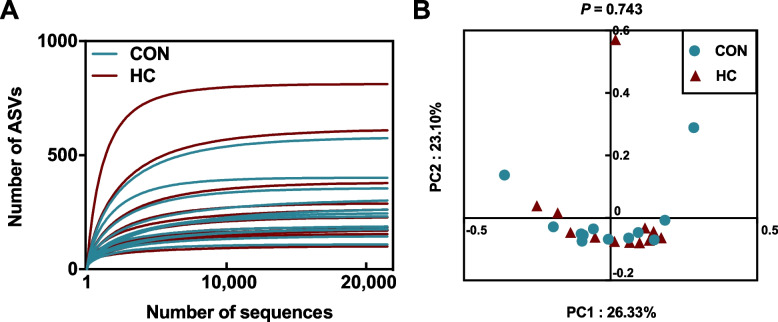
**A** Rarefaction curves based on an amplicon sequence variant (ASV) are shown. Each curve represents one individual sample. **B** Principal component (PC) analysis of Bray-Curtis dissimilarities between the CON and HC diet groups. PERMANOVA results with 999 permutations are shown. CON = conventional diet; HG = high-concentrate diet

The raw sequences were first demultiplexed with an in-house Perl script and the low-quality sequences were filtered out based on the following criteria: the 250 bp reads were truncated at any site receiving an average quality score < 20 over a 10-bp sliding window, discarding the truncated reads that were shorter than 50 bp. The high-quality reads were then merged with a minimum overlap of 10-bp using FLASH v1.2.7 [18]. The sequences were screened to remove chimeras using the Vsearch software (v2.18.0) [19], and further followed by dereplication and amplicon sequence variant (ASV) feature table construction with DADA2 [20] plugin implemented in QIIME 2 v2021.08 [21]. Taxonomic assignment of the ASVs were performed using the naive Bayes classifier [22] trained against the SILVA v138 database [23] that trimmed to match the V3-V4 region sequenced. To control for inter-sample depth variability, all samples were rarefied to the size of the smallest sample (21,465 reads). Rarefaction curves were plotted to guarantee adequate sequencing depth (Fig. 1A). The analyses of α diversity, β diversity, and taxonomic classification were based on the rarefied ASV counts table. Alpha and beta diversity metrics were determined using the plugin q2-diversity in QIIME 2. Beta diversity was measured using Bray-Curtis dissimilarity and visualized with a principal coordinate analysis (PCoA) plot. Statistical significance of the PCoA was conducted by the “adonis” function in the R package “vegan” (v2.5–7) with 999 permutations.

### Milk metabolome analysis

The 24 milk samples used in the HPLC-MS-based metabolomics analyses were prepared as follows. Firstly, the samples were thawed at room temperature and 100 μL of each composite sample from the morning and evening were transferred into a centrifuge tube. Then, all samples were extracted with 300 μL of methanol, and 10 μL of internal standard (2.8 mg/mL, *DL*-o-chlorophenylalanine) was added. Next, the samples were vortexed for 30 s, and incubated for 1 h at −20 °C. Finally, the samples were centrifuged at 13,800 × *g* for 15 min at 4 °C, and 200 μL of the supernatant was transferred to the vial for subsequent HPLC-MS analysis.

The HPLC-MS was performed using an Ultimate 3000LC-Q-Exactive instrument (Thermo, California, USA) incorporating a Hyper gold C18 column (Thermo; 100 mm × 2.1 mm, 1.9 μm), and the column temperature was maintained at 40 °C. The injection volume was 10 μL and the autosampler was maintained at 4 °C. The gradient of mobile phase consisted of A [water + 5% (v/v) acetonitrile + 0.1% (v/v) formic acid] and B [acetonitrile + 0.1% (v/v) formic acid]. The flow rate was 0.3 mL/min. The elution procedure was designated as follows: 5% B for 1 min, 40% B at 2 min, 80% B at 7 min, 95% B at 11 min, and 5% B for 15.5–19.5 min. The mass spectrometric settings for positive and negative ion modes were as follows: heater temperature, 300 °C; sheath gas flow rate, 45 arb; aux gas flow rate, 15 arb; sweep gas flow rate, 1 arb; spray voltage, 3.0 KV/−3.2 KV; capillary temperature, 350 °C; and S-lens RF level, 30%/60%, respectively.

The raw data were conducted with feature extraction and preprocessing using Compound Discoverer 2.0 software (Thermo Scientific). Only ion peak data that were present in ≥ 50% of samples were retained. The main parameters were set as follows: intensity threshold, 300,000; *m/z* range, 70–1050; *m/z* width, 5 ppm; frame time width, 0.2 min; and retention time start and end values were 0.01–19.5 min, respectively. Then, the data were normalized according to the interior label and post-edited in Excel 2010 software. The KEGG database (http://www.genome.jp/kegg) and the Human Metabolome Database (http://www.hmdb.ca) were utilized to identify the metabolites through alignment of the molecular mass data. If the value between theoretical mass and observed mass was less than 10 ppm, the metabolites were reported. And the matched metabolites were further validated by isotopic distribution measurement.

The final metabolites data were imported into SIMCA-P software (Version 13, Umetrics AB, Sweden) for multivariate statistical analysis. The principal component analysis (PCA) and partial least squares-discriminate analysis (PLS-DA) were carried out to explore the differences of the metabolome profile between the two groups. Statistical significance of the PCA was performed using the “adonis” function in the R package “vegan” with 999 permutations. The PLS-DA models were validated based on variation interpretation (R^2^Y) and predictability (Q^2^) of the model in cross-validation and permutation tests with 200 iterations. The differential metabolites between the two groups were screened with a cut-off condition of the Benjamini-Hochberg adjusted *P*-value (*Q*) < 0.05 and the variable importance in the projection (VIP) of PLS-DA model > 1. Fold change (FC, HC vs. CON) and pathway analyses were processed with the web-based tool MetaboAnalyst 5.0 (http://www.metaboanalyst.ca). For the pathway analysis results, differences were regarded significant at *P* < 0.05. The correlation network between the milk composition and the differential milk metabolites was visualized using the Fruchterman-Reingold Algorithm in Gephi 0.9 software (https://gephi.org/) [24].

### Statistical analyses

The milk yield, milk composition, and ruminal pH were analyzed by the linear mixed-effects models (MIXED) procedure of IBM SPSS statistics V25.0 (IBM Corp., Armonk, NY, USA). The treatment (CON or HC), day, and their interaction were treated as fixed factors. The cow was considered a random effect. The milk microbiota and metabolome data were analyzed using the non-parametric Scheirer-Ray-Hare extension of the Kruskal-Wallis test [25], which is a non-parametric analog of ANOVA based on ranked variates with two independent factors (diet and day) plus their interactions. Effects were deemed significant when *P* < 0.05.

## Results

### DMI and ruminal pH

The results for the DMI and rumen pH of the cows were reported previously [16]. Briefly, there was no significant difference in DMI between the CON and HC groups (23.79 vs. 22.40, *P* = 0.524), and the HC feeding resulted in an duration of a rumen pH of < 5.8 of 9.2 h/d in average.

### Milk yield and composition

There were no significant differences in milk yield, 4% fat corrective milk, milk protein ratio, lactose ratio, nor in the yield of milk fat, milk protein, and lactose between the CON and HC groups (*P* > 0.05; Table 2). However, the milk fat percentage was significantly lowered in the HC group compared with the CON group (*P* = 0.034).

**Table 2:** Comparison of milk production and milk composition in cows fed the conventional (CON) and high-concentrate (HC) diets

Item	CON	HC	SEM	*P*-value		
				Diet	Day	Diet × Day
Milk yield, kg/d	19.26	18.90	0.46	0.752	0.427	0.988
4% FCM^a^, kg/d	19.45	18.81	0.47	0.539	0.430	0.989
Milk fat percentage, %	4.31	3.97	0.09	0.034	0.042	0.744
Milk protein percentage, %	3.84	3.79	0.06	0.616	0.055	0.516
Milk lactose percentage, %	5.35	5.20	0.06	0.128	0.001	0.619
Milk fat, kg/d	0.80	0.75	0.02	0.409	0.755	0.887
Milk protein, kg/d	0.71	0.72	0.02	0.922	0.736	0.865
Lactose, kg/d	0.99	0.98	0.03	0.831	0.039	0.915

^a^4% FCM = 0.4 × M + 15 × M × F (*M* Milk yield, *F* Average milk fat percentage, *FCM* Fat corrective milk)

### Structure and composition of the milk bacteria

Across all the 24 milk samples, a total of 1,030,033 high-quality reads pairs were obtained, with an average of 42,918 per sample. The PCoA result based on the Bray-Curtis distance showed that principal coordinates 1 and 2 accounted for 26.33% and 23.10% of the total variance, respectively (Fig. 1B). The samples collected in the HC group did not separate from those in the CON group (PERMANOVA test, *P* = 0.743). The alpha diversity indices of the bacterial communities between the two groups are shown in Table 3, all of which showed no differences between the two groups (*P* > 0.05).

**Table 3:** Comparison of the α-diversity indices of rumen bacterial community based on 16S rRNA gene sequencing

Alpha diversity	Diet		SEM	*P*-value		
	CON	HC		Diet	Day	Diet × Day
Observed ASVs	263	294	35.27	0.751	0.870	0.901
Chao 1	267	297	35.45	0.773	0.907	0.864
Shannon	4.43	4.95	0.21	0.248	0.724	0.873
Simpson	0.12	0.08	0.01	0.248	0.651	0.849

^a^Standardizing sequences depth at 23,465

^b^*ASV* Amplicon sequence variant, *CON* Conventional diet, *HC* High-concentrate diet

Forty-six bacterial phyla were identified among all the samples. Among them, Proteobacteria, Bacteroidetes, Actinobacteria, and Firmicutes were the predominant phyla, representing more than 95% of the bacterial community (97.78% in the CON group and 95.33% in the HC group). There were no significant differences in the relative abundance of all these 4 phyla between the two groups (*P* > 0.05; Table 4).

**Table 4:** Effects of feeding the conventional (CON) or high-concentrate (HC) diet on the relative abundance (%) of rumen bacteria at the phylum level

Phylum	Diet		SEM	*P*-value		
	CON	HC		Diet	Day	Diet × Day
Proteobacteria	72.52	71.47	2.51	0.564	0.462	0.790
Bacteroidota	9.49	11.86	0.84	0.149	0.098	0.631
Actinobacteriota	12.77	8.62	2.33	0.954	0.566	0.349
Firmicutes	3.00	3.38	0.55	0.773	0.129	0.697

At the genus level, 16 predominant taxa whose relative abundance ≥ 1% in at least one group of the CON and HC groups was examined, and the HC group showed a higher abundance of *Labrys* compared to the CON group (*P* = 0.015; Table 5).

**Table 5:** Effects of feeding the conventional (CON) or high-concentrate (HC) diet on the relative abundance (%) of rumen bacteria at the genus level

Genus	Diet		SEM	*P*-value		
	CON	HC		Diet	Day	Diet × Day
*Variovorax*	18.74	13.84	2.09	0.419	0.793	0.438
*Sphingomonas*	9.54	9.79	0.66	0.817	0.595	0.569
*Phyllobacterium*	8.07	9.17	0.69	0.525	0.130	0.425
*Mycobacterium*	10.71	6.49	2.26	0.862	0.523	0.300
*Vibrionimonas*	8.06	8.39	0.86	0.862	0.651	0.961
*Bradyrhizobium*	7.29	8.10	0.57	0.299	0.570	0.444
*Rhodopseudomonas*	5.19	6.45	0.54	0.273	0.761	0.192
*Methylobacterium-Methylorubrum*	5.01	5.59	0.52	0.603	0.898	0.255
*Mesorhizobium*	3.02	3.14	0.18	0.686	0.147	0.661
*Halomonas*	4.05	0.79	1.00	0.141	0.295	0.106
*Allorhizobium-Neorhizobium-Pararhizobium-Rhizobium*	1.65	1.56	0.14	0.729	0.505	0.655
*Labrys*	0.81	1.41	0.12	0.015	0.925	0.300
*Nocardia*	1.09	0.96	0.09	0.686	0.885	0.386
*Chryseobacterium*	0.07	1.12	0.55	0.319	0.135	0.812
*Acinetobacter*	0.30	1.38	0.59	0.707	0.306	0.823
*Candidatus Competibacter*	1.01	0.65	0.21	0.885	0.609	0.605

At the ASV level, we only analyzed the abundant ASVs whose proportions ≥ 0.1% in at least one of the 2 groups (Table S1). The results showed that there were 49 bacterial ASVs co-existed between the 2 groups, accompanied by 1 ASV existing independently in the CON group and 7 ASVs existing independently in the HC group. Compared with the CON group, the HC feeding resulted in a higher relative abundance of ASV44 (genus: *Beijerinckiaceae_*28-YEA-48) and ASV13 (genus: *Labrys*), whereas a lower relative abundance of ASV33 (genus: *Rhodococcus*) (*P* < 0.05; Table S1).

### Identification and general characterization of the milk metabolites

In total, 188 compounds were identified from the milk samples of the CON and HC groups, consisting primarily of fatty acids, lipids, amino acids, sugars, organic acids, nucleotides, and vitamins.

**Fig. 2:**
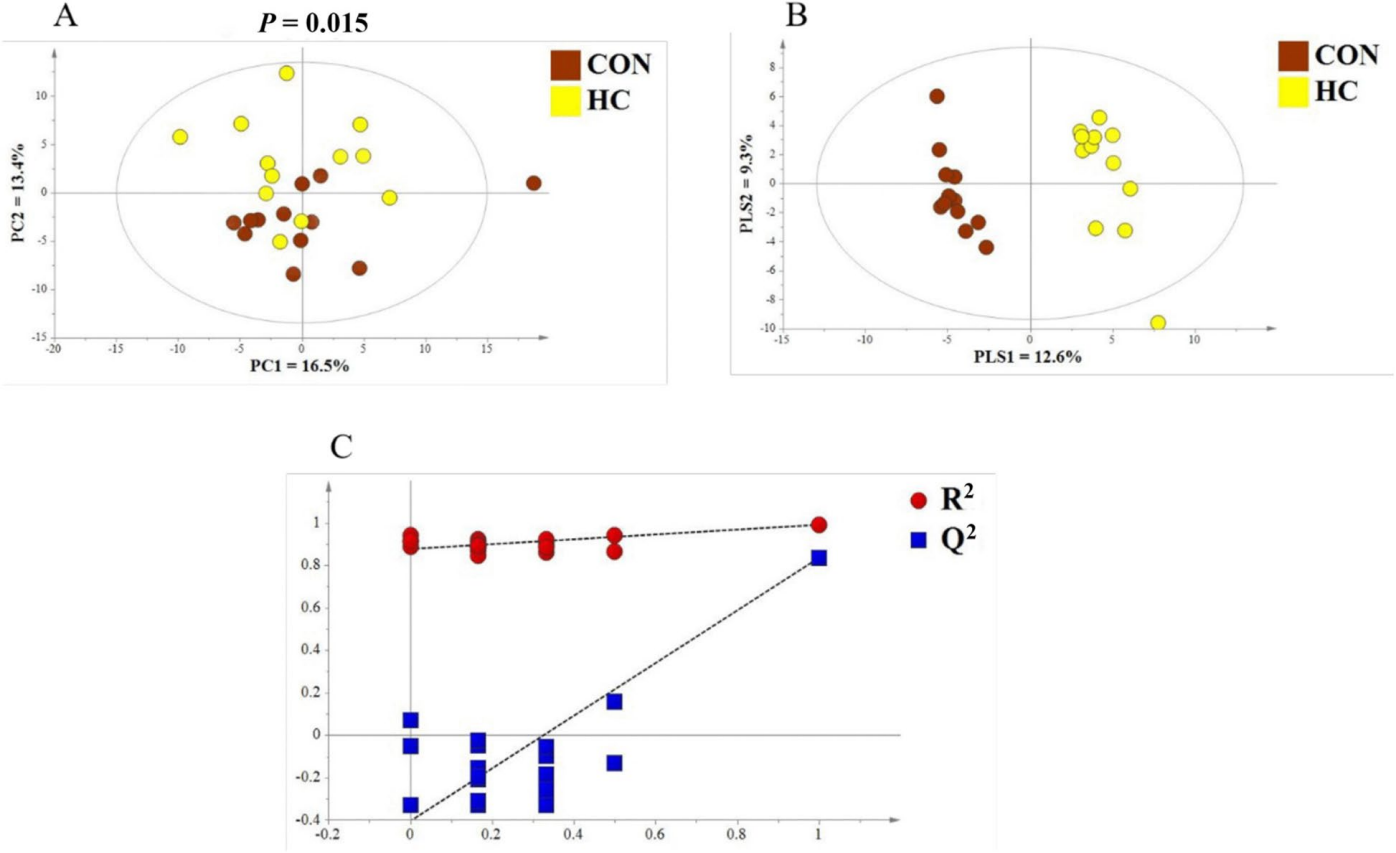
Multivariate analysis of milk metabolome between the conventional (CON) and high-concentrate (HC) diet groups. **A** Principal component (PC) analysis scores plot. PERMANOVA results with 999 permutations are shown. **B** Partial least squares-discriminate analysis scores. PLS1 is the first principal component; PLS2 is the second principal component. **C** Permutation tests plots of 200 iterations. R^2^ and Q^2^ are fitness and predictive power of the model, respectively

The PCA results revealed a distinct difference in milk metabolic profiles between the CON and HC groups (*P* = 0.015 by PERMANOVA; Fig. 2A). Also, the PLS-DA results showed a clear separation between the two groups (Fig. 2B). The validity of the PLS-DA model was evaluated using R^2^Y and Q^2^ [26]. In our study, the R^2^Y and Q^2^ of the PLS-DA score plot was 0.991 and 0.836 respectively, signifying a proper goodness and a high predictive power of the model. Moreover, the Q^2^ intercept value of the permutation test was less than 0, indicating that the model was not overfitted (Fig. 2C).

### Differences in milk metabolites between the CON and HC groups

**Fig. 3:**
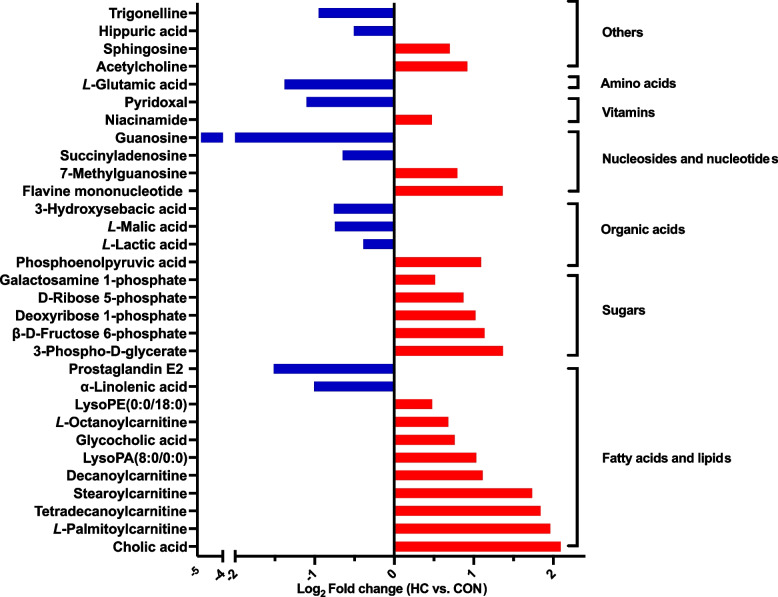
Differential metabolites identified in the milk between the conventional (CON) and high-concentrate (HC) diets feeding groups

**Fig. 4:**
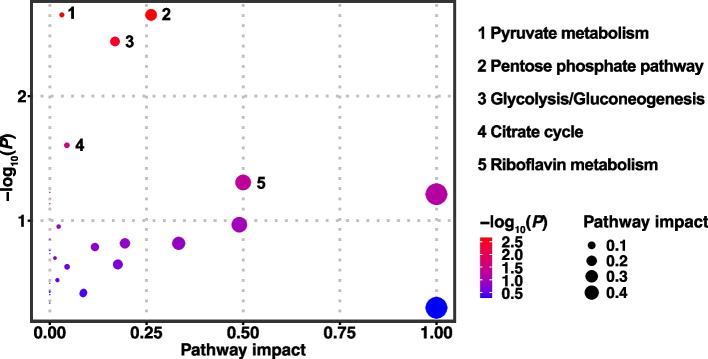
Pathway analysis of KEGG for differential metabolites between the conventional (CON) and high-concentrate (HC) diets feeding groups

With a threshold of VIP > 1 and *Q* < 0.05, a total of 31 differential metabolites were identified between the CON and HC groups. Compared with the CON group, the levels of 20 metabolites were up-regulated in the HC group, including cholic acid, *L*-palmitoylcarnitine, tetradecanoylcarnitine, stearoylcarnitine, decanoylcarnitine, LysoPA(8:0/0:0), glycocholic acid, *L*-octanoylcarnitine, LysoPE(0:0/18:0), 3-phospho-*D*-glycerate, β-*D*-fructose 6-phosphate, deoxyribose 1-phosphate, *D*-ribose 5-phosphate, N-acetyl-α-*D*-galactosamine 1-phosphate, phosphoenolpyruvic acid, flavine mononucleotide, 7-methylguanosine, niacinamide, acetylcholine, and sphingosine. On the contrary, the levels of 11 metabolites were down-regulated in the HC group, including α-linolenic acid (ALA), prostaglandin E2, *L*-lactic acid, *L*-malic acid, 3-hydroxysebacic acid, succinyladenosine, guanosine, pyridoxal, *L*-glutamic acid, hippuric acid, and trigonelline (Fig. 3). Pathway analysis indicated that these 31 metabolites were mainly enriched in 5 key differential metabolic pathways of pyruvate metabolism, pentose phosphate pathway, glycolysis/gluconeogenesis tricarboxylic acid (TCA) cycle, and riboflavin metabolism (*P* < 0.05; Fig. 4).

### Correlation network of the phenotypic traits and the milk metabolites of the cows

**Fig. 5:**
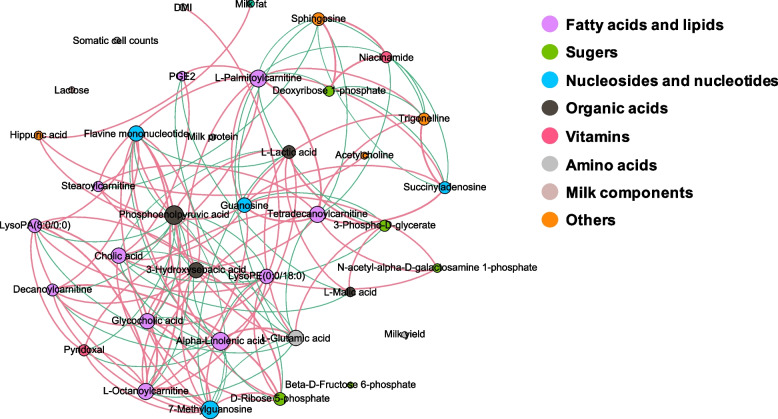
Correlation networks of cow phenotypic traits (DMI, milk yield, and milk composition) and differential milk metabolites based on Spearman’s correlation coefficients (|*r*| > 0.75 and *P* < 0.05). Node size and color corresponds to the correlation degree and substance classification, respectively. Red lines denote positive correlations and green lines denote negative correlations

The correlation network analysis was performed to visualize the relationships of the cows’ phenotypic traits (DMI, milk yield, and milk composition) and the differential milk metabolites (Table S2). Results showed that the correlation network was composed of 37 nodes and 127 edges, which included 77 positive correlations and 50 negative correlations (|*r*| > 0.75 and *P* < 0.05; Fig. 5). Among them, milk fat was positively correlated with hippuric acid, while hippuric acid was positively correlated with phosphoenolpyruvic acid (PEP) and acetylcholine. However, there were no significant correlations between milk yield, milk protein, lactose, and those differential milk metabolites.

## Discussion

In this study, we conducted the comparation of the milk bacterial and metabolic profiles between the cows feeding the CON and HC diets.

The composition and function of the bacterial communities that colonized in the teat apex, teat orifice, and teat canal lining could reflect the status of the udder health [27], and these bacteria play a major role in the development of intramammary infection [28]. Migrating from off-udder sites and the environment are the two major sources of milk bacteria, including from the cows’ teat surface, the milking canal, the milking machines (or other dairy equipment), the bedding material, the feces, the parlor air (stable and milking) and so on [29, 30]. In the current study, the milk bacteria were mainly dominated by Proteobacteria, Actinobacteria, Bacteroidetes, and Firmicutes regardless of diet, which was in line with previous studies on the milk microbiome [31]. At the genus level, there was an increased relative abundance of *Labrys* in the HC group. The *Labrys* belong to the order Rhizobiales, which were abundant in soil-associated environments [32]. Furthermore, the significantly changed abundance of ASV44 (genus: *Beijerinckiaceae_*28-YEA-48) and ASV33 (genus: *Rhodococcus*) between the CON and HC groups were also environment associated taxon [33, 34]. This result suggested that the enriched bacteria in the milk of HC feeding cows were mainly from the external environment, which was consisted with the earlier reported findings [35].

Moreover, one interesting observation from our study was the high abundance of *Mycobacterium* in both the CON and HC groups, and it did not present difference between the 2 groups (Table 5), which implied that its high percentage was not attributed to the HG feeding. Further analyses at the ASV level indicated that this taxon in the present study was composed of only ASV3 and it was aligned to uncultured bacterium at the species level. According to the literature, *Mycobacterium* are not among the most abundant bacterial taxa in studies on bovine milk microbiota [36, 37], and the majority of this genus are opportunistic pathogens [38]. Among them, *Mycobacterium bovis* (*M. bovis*), a pathogen that belongs to the *Mycobacterium tuberculosis* complex, is most commonly reported in researches on cows since it causes zoonotic tuberculosis (TB) disease [39, 40]. Recently, an increasing number of studies reported that *M. bovis* milk contamination is at a relatively wide prevalence as a result of contact between healthy cows and infected cows in modern intensive farming systems [41–43]. Milk-borne transmission of zoonotic TB by *M. bovis* always occurs through the consumption of unpasteurized or poor pasteurized contaminated dairy products [44, 45]. In the United States, about 1%–2% of TB cases are attributable to *M. bovis* infection [46]. Altogether, though we cannot confirm if the high-abundance *Mycobacterium* identified in our study are opportunistic pathogens, it still reminds us that certain pathogens contamination (such as *M. bovis*) can be a latent occurrence in raw milk and pasteurization is vitally important to people’s health.

The most common pathogenic bacteria attributed to the cows’ mastitis are members of genus *Staphylococcus* and *Streptococcus* [47, 48]. Additionally, a recent study revealed that *Stenotrophomonas* might be associated with the development of mastitis in the cows [7]. The possible mechanism for the cows’ mastitis under the HC feeding may due to the translocation of the rumen-derived lipopolysaccharide, which disrupts the blood-milk barrier and eventually leads to the translocation of pathogenic bacteria through blood [7, 49]. However, in the present study, we did not detect *Staphylococcus*, *Streptococcus* or *Stenotrophomonas* in either the CON or the HC groups, which might suggest that the cows did not develop mastitis during our experiment. The inconsistent results might be due to the relatively low concentrate proportion used [6, 7] and the relatively short experiment period adopted [49] in our study, accompanied by the individual variances of the host animals between different studies, which eventually did not cause the translocation of rumen lipopolysaccharide.

Among the 31 differential metabolites between the two groups, the concentrations of all the fatty acylcarnitines were increased in the HC group (Fig. 3). Fatty acylcarnitines are fatty acids esters formed when fatty acyl-CoA are shuttled into the mitochondrion for β-oxidation [50]; and their elevated concentrations here might imply a dysfunction of the lipid metabolism in the HC cows, which could traced to the changes in rumen fermentation (increased propionate concentration, altered lipid metabolism, and so on) caused by the shifts of rumen microbiota profiles (structure, composition, and function) under the HC feeding [16, 51]. The milk metabolite profiles are tightly associated with the coagulation properties of the milk [52, 53]. It revealed that the elevated milk carnitine concentrations would affect the coagulation properties of the milk and the carnitine levels were always lower in good-coagulating milk samples [53]. Moreover, earlier studies demonstrated that higher levels of long-chain acylcarnitines were related with liver diseases, obesity, and type 2 diabetes [54–56]. Therefore, the up-regulated fatty acylcarnitines in the HC diet feeding cows might suggest a lower milk quality in our research.

Compared to the CON group, the level of the milk cholic acid was greatly up-regulated under the HC feeding (FC = 4.27, HC vs. CON). Metabolites in the milk could originate from a variety of different sources, including being secreted by the mammary epithelial cells, being leaked from the damaged somatic cells, being transferred from the blood, or coming from the microbiota metabolism present in the milk [11, 57]. In our earlier study, we found a high level of cholic acid in plasma of the HC group (FC = 2.76, HC vs. CON) [58], so we speculate that the accumulation of cholic acid in the milk might derive from the increased concentration of cholic acid in the blood. Cholic acid is a naturally occurring, primary bile acid that synthesized from cholesterol in the liver [59]. Bile acids are known to have lipid-lowering effects [60], and it has been confirmed that treating gallstone patients with chenodeoxycholic acid could decrease the hepatic very-low-density lipoprotein production and the plasma triglyceride level in clinical trial [61]. The triglyceride concentration was also found to be elevated in plasma of the HC feeding cows in our earlier study (1.508:1.228 mmol/L, HC vs. CON, *P* = 0.014) [58], and it revealed that there was a positive correlation (*Pearson r* = 0.51, *P* = 0.011) between the levels of milk cholic acid and plasma triglyceride.

Besides, the levels of all of the sugars and the corresponding derivatives were up-regulated in the HC group, including 3-phospho-D-glycerate (FC = 2.58, HC vs. CON), β-*D*-fructose 6-phosphate (FC = 2.20, HC vs. CON), deoxyribose 1-phosphate (FC = 2.03, HC vs. CON), *D*-ribose 5-phosphate (FC = 1.83, HC vs. CON), and N-acetyl-α-*D*-galactosamine 1-phosphate (FC = 1.43, HC vs. CON; Fig. 3). Among them, 3-phospho-*D*-glycerate is a biochemically significant metabolic intermediate in glycolysis [62]. Beta-*D*-fructose 6-phosphate and *D*-ribose 5-phosphate, accompanied by the elevated level of an organic acid compound-PEP (FC = 2.13, HC vs. CON) for the HG cows are all involved in both pentose phosphate and glycolysis/gluconeogenesis pathways [63]. Their enrichment indicated that the pentose phosphate pathway and the glycolysis/gluconeogenesis pathway in the mammary gland were affected by the HC diet feeding. Deoxyribose-1-phosphate is a proangiogenic paracrine stimulus released by platelets and macrophages, which acts on endothelial cells to drive tissue repair [64]. Hence, the increased level of deoxyribose-1-phosphate in the HC group might hint a potential injury of the mammary epithelial cells.

In contrast, the HG diet feeding resulted in a decreased concentration of milk ALA (FC = 0.50, HC vs. CON). The ALA is an important precursor to the synthesis of docosahexaenoic acid, which is a major contributor to the visual acuity and brain development [65]. Furthermore, it has been reported that when the cows were fed an ALA-enriched diet, the expression of some local and systemic pro-inflammatory markers in vivo would be down-regulated [66]. Therefore, the decreased concentration of ALA in the milk of the HC feeding cows might reduce the levels of the beneficial ingredients in the milk, which finally impact the milk quality. Additionally, the decreased level of *L*-malic acid (FC = 0.76, HC vs. CON) in milk with the HC feeding might be explained by the changes in the energy metabolism. In detail, SARA could cause an impaired TCA cycle function, which in turn results in active secretion or leakage of *L*-malic acid from the mammary epithelial cells into the milk [67]. Glutamic acid can be converted into alpha-ketoglutarate, which was a fuel for the TCA cycle [68]. The declined level of *L*-glutamic acid in the HC group (FC = 0.38, HC vs. CON) might also be a mirror of the impaired TCA cycle.

As for the other differential metabolites between the 2 groups, the decreased levels of succinyladenosine (FC = 0.64, HC vs. CON) and guanosine (FC = 0.04, HC vs. CON), and the increased levels of flavine mononucleotide (FC = 2.57, HC vs. CON) and 7-methylguanosine (FC = 1.73, HC vs. CON) implied a dysfunction of nucleotide metabolism in the mammary gland of the HC cows. Hippuric acid is a benzoilglycine, a compound of benzoic acid and glycine, which could lead to the detoxication of benzoic acid and benzoates [69]. In the present study, we found that the concentration of hippuric acid was decreased in the HC cows (FC = 0.70, HC vs. CON) and it was closely correlated with milk fat, PEP and acetylcholine. Up to now, there is not any study that reports the association between hippuric acid and milk fat. However, hippuric acid has been found to be associated with diet, and it was proposed as a biomarker for organic feeding [70, 71]. The PEP is involved in the TCA cycle, therefore, the decreased concentration of hippuric acid in the HC group might mirror certain alterations in the energy metabolism of the cows. At the same time, it echoes the above-discussed result of the reduced concentration of *L*-malic acid in the HC cows which might be caused by the impaired TCA function. Acetylcholine is found in mesothelial, endothelial, glial, circulating blood cells (platelets, mononuclear cells), and alveolar macrophages [72]. Non-neuronal acetylcholine appears to be involved in the regulation of some important cell functions, such as mitosis, trophic functions, automaticity, locomotion, ciliary activity, cell-cell contact, cytoskeleton, and barrier and immune functions [73]. Hence, the increased level of acetylcholine in the HC cows (FC = 1.89, HC vs. CON) and its association with the decreased level of hippuric acid might imply a feedback regulation of the mammary gland against the damages caused by SARA.

## Conclusions

The 16S rRNA gene sequencing and HPLC-MS methods were combined to analyze the differences in milk bacterial and metabolic profiles between the CON and HC groups. Taken together, our results indicated that the bacterial community of the milk was primarily dominated by Proteobacteria, Actinobacteria, Bacteroidetes, and Firmicutes at the phylum level, and the differential taxon between the 2 groups were mainly from the external environment. Besides, our results revealed a comprehensive profiling of the milk metabolome associated with SARA and suggested that HC diet feeding led to a decline of the milk quality. In addition, there were no direct or indirect utilization or productive relationships found between the milk bacteria and metabolites through our results. All these findings are beneficial to our subsequent research to explore the diagnostic biomarkers in the milk of cows with SARA and provide us with a new sight to further explore the occurrence and harms of SARA.

## Supplementary Information

**Additional file 1: Table S1.** Effects of feeding the conventional (CON) or high-concentrate (HC) diet on the relative abundance (%) of rumen bacteria at the amplicon sequence variant (ASV) level.

**Additional file 2:** **Table S2.** Correlation networks of cow phenotypic traits (DMI, milk yield, and milk composition) and differential milk metabolites based on Spearman’s correlation coefficients.

## Data Availability

Raw reads of milk 16S rRNA gene sequencing were deposited in NCBI SRA database under accession number PRJNA736538.

## Abbreviations

ALA: α-linolenic acid
ASV: Amplicon sequence variant
CON: Conventional
DMI: Dry matter intake
FC: Fold change
HC: High-concentrate
HPLC-MS: High-performance liquid chromatography-mass spectrometry
PCA: Principal component analysis
PCoA: Principal coordinate analysis
PEP: Phosphoenolpyruvic acid
PLS-DA: Partial least squares-discriminate analysis
SARA: Subacute rumen acidosis
TB: Tuberculosis
TCA: Tricarboxylic acid
VIP: Importance in the projection
